# Supplementary material for: MetaRibo-Seq measures translation in microbiomes
Source: Nat Commun. 2020 Jun 29;11:3268. doi: 10.1038/s41467-020-17081-z (PMC7324362; doi:10.1038/s41467-020-17081-z)
Supplement: Supplementary file 10 — Supplementary Data 7 [file 41467_2020_17081_MOESM10_ESM.zip › File2/Confidence_High_Taxonomy/112639_out.krona.html]

Javascript must be enabled to view this page.

members
magnitude
magnitudeUnassigned
count
unassigned
taxon
rank

112639\_out

11


SRS013098\_contig\_number\_contig-100\_27480.54065SRS015890\_contig\_number\_contig-100\_14750.14751SRS018427\_contig\_number\_contig-100\_13006.13006SRS105153\_contig\_number\_11885
4

2
superkingdom
7

7
1239
phylum

7
186801
class


SRS018984\_contig\_number\_5494SRS148721\_contig\_number\_contig-100\_16791.120997
order
2
7
186802

4
541000
family

4

SRS019787\_contig\_number\_8207SRS024388\_contig\_number\_contig-100\_1284.61999SRS104311\_contig\_number\_21943SRS146764\_contig\_number\_21972
1952302
species

species
1950913

SRS015960\_contig\_number\_contig-100\_12206.12206
1
